# Microglial CX_3_CR1 promotes adult neurogenesis by inhibiting Sirt 1/p65 signaling independent of CX_3_CL1

**DOI:** 10.1186/s40478-016-0374-8

**Published:** 2016-09-17

**Authors:** Sabine Sellner, Ricardo Paricio-Montesinos, Alena Spieß, Annette Masuch, Daniel Erny, Laura A. Harsan, Dominik v. Elverfeldt, Marius Schwabenland, Knut Biber, Ori Staszewski, Sergio Lira, Steffen Jung, Marco Prinz, Thomas Blank

**Affiliations:** 1 Institute of Neuropathology, University of Freiburg, Breisacher Str. 64, D-79106 Freiburg, Germany; 2 Department of Psychiatry and Psychotherapy, Freiburg, Germany; 3 Department of Radiology, Medical Physics, University Medical Center Freiburg, Freiburg, Germany; 4 Department of Neuroscience, University Medical Center Groningen, University of Groningen, Groningen, The Netherlands; 5 Immunology Institute, Icahn School of Medicine at Mount Sinai, New York, USA; 6 Department of Immunology, Weizmann Institute of Science, Rehovot, Israel; 7 BIOSS Center for Biological Signaling Studies, University of Freiburg, Freiburg, Germany; 8 Present Address: Laboratory of Experimental Stroke Research, Institute for Stroke and Dementia Research (ISD), University of Munich Medical Center, 81377 Munich, Germany; 9 Present Address: Department of Neuroscience, Max Delbrück Center for Molecular Medicine (MDC), Berlin-Buch, Robert-Rössle-Str. 10, 13092 Berlin, Germany; 10 Present Address: Cluster of Excellence NeuroCure, Neuroscience Research Center, Charité-Universitätsmedizin Berlin, Charitéplatz 1, 10117 Berlin, Germany; 11 Present Address: Institute of Clinical Chemistry and Laboratory Medicine, University Medicine Greifswald, Ferdinand-Sauerbruch-Str., 17475 Greifswald, Germany

**Keywords:** Microglia, Water maze, Morphometry, Adult neurogenesis, Sirtuin 1, NF-kB p65 acetylation

## Abstract

Homo and heterozygote *cx3cr1* mutant mice, which harbor a green fluorescent protein (EGFP) in their *cx3cr1* loci, represent a widely used animal model to study microglia and peripheral myeloid cells. Here we report that microglia in the dentate gyrus (DG) of *cx3cr1*^−/−^ mice displayed elevated microglial sirtuin 1 (SIRT1) expression levels and nuclear factor kappa-light-chain-enhancer of activated B cells (NF-kB) p65 activation, despite unaltered morphology when compared to *cx3cr1*^+/−^ or *cx3cr1*^+/+^ controls. This phenotype was restricted to the DG and accompanied by reduced adult neurogenesis in *cx3cr1*^−/−^ mice. Remarkably, adult neurogenesis was not affected by the lack of the CX_3_CR1-ligand, fractalkine (CX_3_CL1). Mechanistically, pharmacological activation of SIRT1 improved adult neurogenesis in the DG together with an enhanced performance of *cx3cr1*^−/−^ mice in a hippocampus-dependent learning and memory task. The reverse condition was induced when SIRT1 was inhibited in *cx3cr1*^−/−^ mice, causing reduced adult neurogenesis and lowered hippocampal cognitive abilities. In conclusion, our data indicate that deletion of CX_3_CR1 from microglia under resting conditions modifies brain areas with elevated cellular turnover independent of CX_3_CL1.

## Introduction

CX_3_CR1 is a seven transmembrane domain receptor coupled to G_i_ and G_z_ subtypes of G proteins, activation of which is linked to several intracellular second messengers like phosphoinositide 3-kinase (PI3K), protein kinase B (AKT) and nuclear factor kappa-light-chain-enhancer of activated B cells (NFkB) [1]. CX_3_CR1 is prominently expressed by monocytes, subsets of NK and dendritic cells, and the brain microglia [2]. Surprisingly little is known on which intracellular signaling pathways in microglia are affected by the lack of CX_3_CR1. Microglial cells are fundamentally distinct from other brain cells in that they are derived from primitive myeloid progenitors that arise during embryogenesis [3–5]. They represent the resident phagocytic cells in the brain, taking part in immune-mediated defense mechanisms and clearing cell debris [6]. Microglial cells are constantly surveying their surroundings and are implicated in synaptic pruning, during both development and throughout adulthood, and therefore believed to play a role in regulating homeostatic neuronal synaptic plasticity [7, 8]. Neurons and microglia are thought to communicate with one another through neuronal expression of the CX_3_CR1 ligand CX_3_CL1 (or fractalkine). CX_3_CL1 is expressed at the cell membrane of selected neurons and binds to and activates CX_3_CR1 receptors on microglia [9, 10]. CX_3_CL1 exists in two distinct forms: a full-length membrane-bound form and a shed form that contains the N-terminal chemokine domain. The shed chemokine domain of CX_3_CL1 acts, when cleaved, as a signaling molecule and can bind microglial-expressed CX_3_CR1 receptors [11], whereas its membrane-bound mucin stalk can serve as a cell adhesion molecule [12]. CX_3_CL1 is abundantly expressed in the granular cell layer of the rat dentate gyrus (DG), where addition of recombinant CX_3_CL1 reverses age-related decline in adult neurogenesis [13]. Both, *cx3cr1*^−/−^ and *cx3cr1*^+/−^ mice display reduced hippocampal neurogenesis compared with wild-type controls [13, 14]. However, it is not clear to what extent CX_3_CL1 is mandatory for proliferation and neurogenesis. In addition to the already mentioned reduced hippocampal neurogenesis, *cx3cr1*^−/−^ mice were reported to exhibit excessive hippocampal IL-1β expression and either enhanced [15] or attenuated long term potentiation (LTP) [14] resulting in improved [15] or impaired cognitive functions [13, 14]. It is important to mention that Maggi et al. were using exclusively female mice (3 months of age). Rogers et al. and Bachstetter et al. performed all experiments with male mice, 3 months and 4 months of age, respectively. In addition to these conflicting results, little is known about the intracellular signaling cascades activated by CX_3_CR1 deficiency, which might impact synaptic plasticity and cognition. One of these pathways could include the NF-kB signaling pathway, which may trigger microglial activation and induce the release of inflammatory factors including IL-1β, as seen following irradiation [16], during normal aging [17] or neurodegeneration [18]. Along these lines, sirtuin 1 (SIRT1), a member of the sirtuin family, might be modulated in microglia by the lack of CX_3_CR1 because it can suppress inflammatory responses by inhibiting NF-kB signaling [19, 20].

Here we investigated the consequences of the microglial CX_3_CR1 deletion on cell morphology, activation of the NF-kB signaling pathway, the expression of SIRT1, interference with neuroblasts, immature neurons and cognition. Our findings indicate that, under brain homeostasis, hippocampal microglia from *cx3cr1*^−/−^ mice are very similar, if not identical, to microglia from adult wild-type animals in contrast to the situation in newborns or during development. However, hippocampal *cx3cr1*^−/−^ microglia show activation of SIRT1 and the NF-kB pathway in areas of adult neurogenesis. We found that manipulation of SIRT1 activation in *cx3cr1*^−/−^ mice directly impacts cognitive performance, while the same treatment had no detectable effect on cognition in wild-type littermates.

## Materials and methods

This article does not contain any studies with human participants performed by any of the authors.

### Mice

For all animal experiments “Principles of laboratory animal care” (NIH publication No. 86–23, revised 1985) were followed. All experiments were approved by the Federal Ministry for Nature, Environment and Consumers’ Protection of the states of Baden-Württemberg (G12/71 and G11/50), and were carried out in accordance to the respective national, federal, and institutional regulations. Adult, 8–12 weeks old, male mice were used for the experiments. Mice were group housed up to five per cage with 12 h light/dark cycle with lights on at 6 a.m. Food and water were available ad libitum. *cx3cr1*^gfp/gfp^ on a C57BL/6 J background were obtained from the Jackson Laboratory. *cx3cl1*^−/−^ mice were described previously [21]. In all experiments, wild type littermates were used as controls.

### Morris Water Maze (MWM) test

The MWM was used to measure spatial learning and memory [22]. The apparatus consisted of a black plastic pool with a diameter of 120 cm. A black escape-platform (square, 10 × 10 cm) was located 1.0 cm below (hidden) the water surface. The temperature of the water was kept constant throughout the experiment (20 ± 0.5 °C), and a 10 min recovery period was allowed between the training trials. The training consisted of 6 consecutive days of testing, with four trials per day. If the mouse failed to find the escape platform within the maximum time (60 s), the animal was placed on the platform for 10 s by the experimenter. During the first 6 days of testing, the mice were trained with a hidden platform. The platform location was kept constant, and the starting position varied between four constant locations at the pool rim. Mice were placed in the water with their nose pointing toward the wall at one of the starting points in a random manner. On the 7^th^ day, the platform was removed, and the mice were allowed to swim for 60 s to determine their search bias. On testing day 8, mice were trained to find a visible platform, which had a 10 cm high pole with a white flag and was changed every trial to a new position. Timing of the latency to find the visible platform was started and ended by the experimenter. A computer running the *BIOBSERVE* software (BIOBSERVE) analyzed all variables of the MWM test. All behavioral experiments were carried out in a double-blind fashion and mice were tested in random order.

### Mouse treatments

EX527 (cat. no. 2780) was purchased from Tocris Bioscience (Bristol, UK), dissolved in 4 % DMSO/10 % cyclodextrin in PBS and administered intraperitoneally (200 μl, i.p.) at a concentration of 250 ng/μL once daily for 21 days [23]. Resveratrol (cat. no. R5010, Sigma-Aldrich, St. Louis, USA) was dissolved in PBS and i.p. injected once daily over a period of 20 days at a concentration of 2 μg/μL [24].

### BrdU injections

Adult mice received two intraperitoneal (i.p.) BrdU-injections (Sigma-Aldrich, St. Louis, MO, USA, 50 mg/kg in 0.9 % NaCl, 8 h apart) over 3 days [25]. Animals were perfused 3 weeks after the last BrdU injection.

### Histology

Histology was performed as described previously [26, 27]. Mice were perfused intracardially with ice-cold PBS, pH 7.4, and 4 % paraformaldehyde/PBS, pH 7.4. Brains were removed, postfixed overnight in the same fixative, and paraffin embedded or frozen at −20 °C. Coronal sections of 20 μm thickness were cut, deparaffinized in xylene, and blocked with 10 % goat serum/PBS plus 0.2 % Triton X-100. 30 μm thick free-floating coronal brain sections were used from perfusion-fixed frozen brain. Sections were incubated with primary antibody against: Iba-1 (24 h, dilution 1:500 at 4 °C, cat. no. 019-19741, Wako, Osaka, Japan) [28] to detect microglia, NeuN (24 h, dilution 1:200 at 4 °C, cat. no. MAB377, Merck Millipore, Darmstadt, Germany) [29] to detect neurons, DCX (48 h, dilution 1:250 at 4 °C, C-18, sc-8066, Santa Cruz Biotechnology, Dallas, USA) [30] to detect neuroblasts and immature neurons, BrdU (24 h, dilution 1:200 at 4 °C, cat. no. OBT0030, ABD, Serotec, Raleigh, NC, USA) [30] to detect BrdU incorporation, Lamp2 (24 h, dilution 1:250 at 4 °C, cat. no. ab13524, Abcam, Cambridge, UK) [28] to label lysosomes and late endosomes, Ki67 (24 h, dilution 1:800 at 4 °C, cat. no. ab15580, Abcam, Cambridge, UK) [31] to detect proliferating cells, CD11b (48 h, dilution 1:200 at 4 °C, cat. no. ab8878, Abcam, Cambridge, UK) [32] to label ramified parenchymal microglia, and activated caspase-3 (24 h, dilution 1:100 at 4 °C, cat. no.9661, Cell Signaling Technology, Danvers, MA, USA) [33], which is involved in the activation cascade of caspases responsible for apoptosis execution. As secondary antibodies we used: Alexa-Fluor-647–conjugated secondary antibody (cat. no. A31573, Life technologies, Waltham, USA) incubated for 2 h, dilution 1:500, at room temperature, Alexa-Fluor-568–conjugated secondary antibody (cat. no. A11011, Life technologies) was incubated at a dilution of 1:500 for 2 h at 20–22 °C, and Alexa Fluor 488–conjugated secondary antibody (cat. no. A11008, Life technologies), which was added with a dilution of 1:500 overnight at 4 °C. Nuclei were counterstained with 4,6-diamidino-2-phenylindole (DAPI, cat. no. 236276, Boehringer). Images were either taken using the BZ-9000 Biorevo microscope (Keyence, Neu-Isenburg, Germany) or the Olympus FluoView 1000 confocal laser scanning microscope (Olympus, Tokyo, Japan). Integrated fluorescence intensities were measured using Image J 1.4 software [34].

### Cell quantification

Cells were counted using the optical fractionator, a method for unbiased stereological analysis. This method was performed using a computer-assisted image analysis system, consisting of a Leica DMRB/DIC fluorescence microscope equipped with a computer-controlled motorized stage, a video camera, and the Stereo Investigator software (MicroBrightField, Williston, VT). The number of positive cells throughout the rostrocaudal extent of the dentate gyrus was counted with a coded one-in-16 series for frozen sections (40 μm). We used a modified version of the optical fractionator method as reported previously [35–37]. The total numbers of positive cells were multiplied by 16 and reported as total number of cells per dentate gyrus. For immunocytochemical analysis of paraffin sections (30 μm), serial coronal sections were collected spanning the rostrocaudal extent of the hippocampus. For quantification, every 12^th^ section was selected. Every positive cell was counted on these sections, and, to obtain the relative total number of cells in the dentate gyrus, these counts were multiplied by 12 to account for the sampling frequency [25]. In control experiments we could not detect significant differences in cell numbers, when samples were quantified by both quantitation methods.

### Three-dimensional reconstruction of microglia

40-μm coronal cryo sections from adult brain tissue were stained with anti-Iba-1 (cat. no. 019-19741, Wako) [28] for 48 h (dilution 1:500 at 4 °C), followed by staining with an Alexa Fluor 488–conjugated secondary antibody (cat. no. A11008, Life technologies), which was added with a dilution of 1:500 overnight at 4 °C. Nuclei were counterstained with DAPI. Imaging was performed on an Olympus Fluoview 1000 confocal laser scanning microscope (Olympus) using a 20× 0.95 NA objective. Z stacks were imaged with 1.14-μm steps in z direction, 1024 × 1024 pixel resolution were recorded and analyzed using IMARIS software (Bitplane). Four cortical cells were reconstructed per analyzed mouse.

### Laser microdissection

Microdissection of microglia was performed using a Zeiss PALM MicroBeam as described previously, with modification [29]. Fast immunochemistry of serial sections was performed with CD11b antibodies (Serotec). Immunostained sections were counterstained with DAPI to facilitate the identification of individual cells. RNA was isolated with the Rneasy Micro Plus Kit (Qiagen), and reverse transcription (RT), preamplification, and real-time PCR were performed using Applied Biosystems reagents according to the manufacturer’s recommendations. The primer pairs were used as described previously [29].

### Microglia isolation and flow cytometry

Adult microglia cells were harvested from dissected hippocampal tissue using density gradient separation and were prepared as described before [27]. In short, samples were stained for CD11b and CD45 (eBioscience, BD Pharmingen) [4]. Cell suspensions were acquired on a FACS Canto II (Becton Dickinson) or cell populations were sorted with a MoFlo Astrios (Beckman Coulter) and further processed. Data were analyzed using FlowJo software (Tree Star).

### Gene expression analysis

FACS-sorted microglial cell populations were collected directly in cell lysis buffer and subsequently RNA was isolated with the Arcturus Pico Pure RNA Isolation Kit (Life Technologies) according to the manufacturer’s protocol. Reverse transcription and real-time PCR analysis were performed using high capacity RNA-to-cDNA-Kit and Gene Expression Master Mix reagents (Applied Biosystems) according to the manufacturer’s recommendations. RT-PCRs were analyzed with a LightCycler 480 (Roche) and carried out as described previously [26, 38]. The following primers were used: HDAC1: forward 5′-TCACCATGGCGATGGCGTGG-3′, reverse 5′-TGCCGTCTCGCAGTGGGTAGT-3′; HDAC2: forward 5′-ACAGACCCCAAAGGAGCCAAGT-3′, reverse 5′-GCCAATGTCCTCAAACAGGGAAGG-3′; HDAC3: forward 5′-CCTCTGACTTCCTTCTGGGTTCCCC-3′, reverse 5′-TCACACCCTCTCCTCCTTGCCA-3′; HDAC4: forward 5′-TCGGGCACAGTCCTCCCCAG-3′, reverse 5′-TGCGTCCACGGATGCACTCA-3′; HDAC 5: forward 5′-CCTGTCCCGTCCGTCTGTCTGTT-3′, reverse 5′-GCCATCTGCCGACTCGTTGGGAGA-3′; HDAC6: forward 5′-CTGGCGGACTAGAAAGAGCCTTTCC-3′, reverse 5′-GGGGTGACTGGGGATTGTGCC-3′; HDAC7: forward 5′-CCCACATCAGATAACCCAACCACAG-3′, reverse 5′-CTGGAGGGCAGGGGAGCCTTA-3′; HDAC8: forward 5′-CTCGCGGACGGTTGGAAGTGG-3′, reverse 5′-AGTGGACCATACTGGCCCGTT-3′; HDAC9: forward 5′-AGCTTCTCGTGGCTGGTGGA-3′, reverse 5′-CGATTCAGGGGTCGGTGGCG-3′; HDAC10: forward 5′-TTGCTGCAGGTGGCTGCTCC-3′, reverse 5′-CTCGGGCCATGGTTCGCTGG-3′; HDAC11: forward 5′-CAGCCCAGCGGGCATTGTGA-3′, reverse 5′-TCTGTGCCGAGACGCAGGGA-3′; Sirt1: forward 5′-AGCTGGGGTTTCTGTCTCCTGTGG-3′, reverse 5′-ACGGCTGGAACTGTCCGGGAT-3′; Sirt2: forward 5′-CTCGGCCTCTTCTTGTTTCCGCT-3′, reverse 5′-CGAGTCTGAATCGGTCCGGCTC-3′; Sirt3: forward 5′-CGCTTGACCCTCTAGGCGCC-3′, reverse 5′-CCTTCTCCCACCTGTAACACTCCCG-3′; Sirt4: forward 5′-ACGGATGCATGCACAGAGTCCTG-3′, reverse 5′-GAACACGTCGCCGTCGGGAG-3′; GAPDH: forward 5′-TCCTGCACCACCAACTGCTTAGCC-3′, reverse 5′-GTTCAGCTCTGGGATGACCTTGCC-3′.

Mice underwent diffusion tensor imaging (DTI) examination at 156^2^ × 250 μm^3^ spatial resolution with a cryogenic cooled resonator (CCR) at ultrahigh field (7 T) as described previously [39, 40]. Diffusion images were acquired along 30 gradient directions plus 5 references without diffusion encoding with a total acquisition time of 35 min. Fractional anisotropy (FA) maps were statistically compared by whole brain-based spatial statistics (WBSS) at the group level vs. wt controls.

### Sirtuin 1 activity assay

To quantify sirtuin 1 (Sirt1) activity, nuclear extracts from sorted microglia (pooled from hippocampi of three mice per group) were prepared. Nuclear extracts were used to measure deacetylase activity of an acetylated histone using Epigenase Universal SIRT Activity/Inhibition Assay Kit (Epigentek, Farmingdale, NY) [41].

### Statistical analysis

Statistical analysis was performed using GraphPad Prism (GraphPad Software, Version 6.0, La Jolla, USA). In general, chosen sample sizes are similar to those reported in previous publications [38]. All data were tested for normality applying the Kolmogorov-Smirnov test. If normality was given, an unpaired *t* test was applied. If the data did not meet the criteria of normality, the Mann–Whitney *U* test was applied. To test for effects of treatment or genotype a two-factorial analysis of variance (ANOVA) with *post hoc* Bonferroni test or Tukey-Kramer HSD test was applied. Differences were considered significant when *p* < 0.05. Number of animals per group is given as “n”. To obtain unbiased data, experimental mice were all processed together by technicians and cell quantifications were performed blinded by two scientists independently and separately.

## Results

### Efficient adult hippocampal neurogenesis relies on the presence of CX_3_CR1, but is independent on the presence of CX_3_CR1

**Fig. 1:**
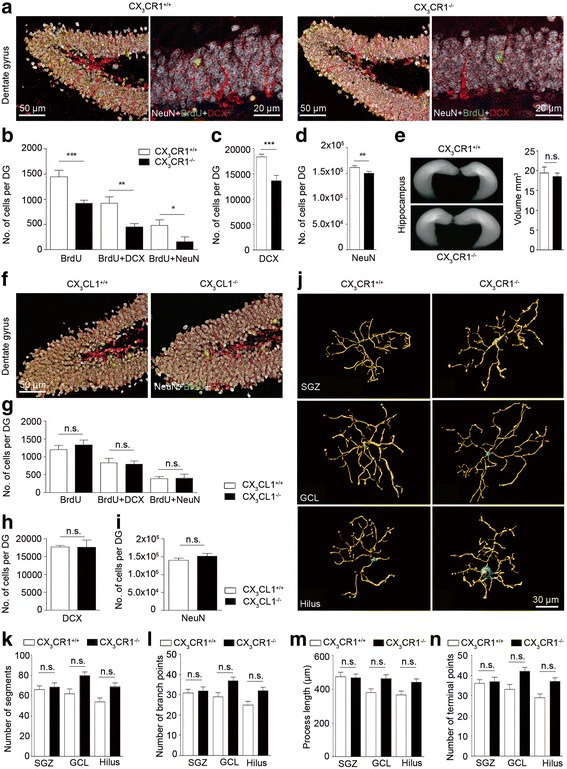
DCX^+^ cell numbers in the subgranular zone (SGZ) of the dentate gyrus (DG) are regulated by the fractalkine receptor, CX_3_CR1, but not by its ligand, fractalkine, under non-stimulated conditions. **a** Representative immunofluorescent images of coronal DG sections from *cx3cr1* ^+/+^ (left) and *cx3cr1* ^−/−^ (right) mice stained for the neuronal marker NeuN (*white*), the proliferation marker bromdesoxyuridin (BrdU, *green*) and DCX (*red*). **b** Quantification of the number of BrdU (*p* < 0.0001, two-tailed Student’s *t*-test, *cx3cr1* ^+/+^ (*n* = 14) vs. *cx3cr1* ^−/−^ (*n* = 13)) BrdU + DCX (*p* = 0.0029, two-tailed Student’s *t*-test, *cx3cr1* ^+/+^ (*n* = 13) vs. *cx3cr1* ^−/−^ (*n* = 13)) and BrdU + NeuN (*p* < 0.0444, two-tailed Student’s *t*-test, *cx3cr1* ^+/+^ (*n* = 10) vs. *cx3cr1* ^−/−^ (*n* = 10)) positive cells in DG revealed lower cell numbers in *cx3cr1* ^−/−^ mice than in *cx3cr1* ^+/+^ mice. Similarly, *cx3cr1* ^−/−^ mice had reduced total numbers of DCX^+^ (*p* < 0.0001, two-tailed Student’s *t*-test, *cx3cr1* ^+/+^ (*n* = 14) vs. *cx3cr1* ^−/−^ (*n* = 13)) (**c**) and NeuN^+^ cells when compared to *cx3cr1* ^+/+^ controls (*p* = 0.0048, two-tailed Student’s *t*-test, *cx3cr1* ^+/+^ (*n* = 9) vs. *cx3cr1* ^−/−^ (*n* = 9)) (**d**) even though the overall hippocampal volume was not different in both mouse lines (*p* = 0.6362, two-tailed Student’s *t*-test, *cx3cr1* ^+/+^ (*n* = 4) vs. *cx3cr1* ^−/−^ (*n* = 4)) (**e**). **f** Representative immunofluorescent images of coronal DG sections from *cx3cl1* ^+/+^ (left) and *cx3cl1* ^−/−^ (right) mice stained with antibodies against NeuN (*white*), BrdU (*green*) and DCX (*red*). **g** In the DG of both mouse strains cell numbers for BrdU, BrdU + DCX and BrdU + NeuN positive cells where not statistically different. There was further no difference in the cell numbers quantified for DCX^+^ (**h**) and NeuN^+^ cells (**i**) (*p* > 0.05, two-tailed Student’s *t*-test, *cx3cl1* ^+/+^ (*n* = 9) vs. *cx3cl1* ^−/−^ (*n* = 9)). **j** 3D-reconstruction of ionized calcium-binding adapter molecule 1 (Iba-1)-labeled microglia from *cx3cr1* ^+/+^ (*left*) and *cx3cr1* ^−/−^ (*right*) mice localized in the SGZ (*top row*), granular cell layer (GCL, middle row) and hilus (bottom row) of the DG. There were no significant morphometric differences in microglia of both mouse lines after quantifying **k** number of segments, **l** number of branch points, **m** process length and **n** number of terminal points (*p* > 0.05, two-tailed Student’s *t*-test, *cx3cr1* ^+/+^ (*n* = 25–31 cells from 4 animals) vs. *cx3cr1* ^−/−^ (*n* = 31–37 cells from 4 animals)). One representative experiment of two is shown. Bars represent mean ± SEM; n.s., not significant. ***p < 0.001; **p < 0.01; *p < 0.05

**Fig. 2:**
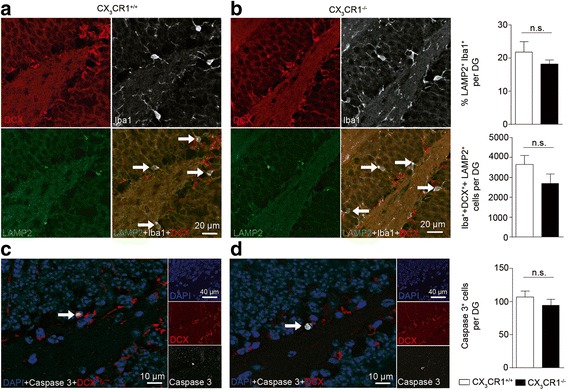
Reduced cell number of DCX^+^ cells in the DG of *cx3cr1* ^−/−^ mice is not due to increased microglial phagocytosis or DCX^+^ cell apoptosis. **a** Representative coronal DG sections from *cx3cr1* ^+/+^ (**a**) and *cx3cr1* ^−/−^ mice (**b**) immunostained for the microglia marker Iba1 (*white*), the phagocytosis marker lysosome-associated membrane protein 2 (LAMP2, *green*) and DCX (*red*) labeling proliferating cells and young developing neurons. Quantification of LAMP2^+^Iba1^+^ cells (top right) (*p* = 0.3957, two-tailed Student’s *t*-test, *cx3cr1* ^+/+^ (*n* = 12) vs. *cx3cr1* ^−/−^ (*n* = 8)) or of Iba1^+^DCX^+^LAMP2^+^ cells (middle right) (*p* = 0.1551, two-tailed Student’s *t*-test, *cx3cr1* ^+/+^ (*n* = 8) vs. *cx3cr1* ^−/−^ (*n* = 8)) showed no significant difference between both mouse lines. Similar numbers of apoptotic cells were detected by an active caspase 3 marker (*white*) co-stained with DAPI (*blue*) and DCX (*red*) in the DG of *cx3cr1* ^+/+^ (**c**) and *cx3cr1* ^−/−^ mice (**d**) (*p* = 0.3406, two-tailed Student’s *t*-test, *cx3cr1* ^+/+^ (*n* = 13) vs. *cx3cr1* ^−/−^ (*n* = 13)) as depicted in the bar graph (*bottom right*). One representative experiment of two is shown. Bars represent mean ± SEM; n.s., not significant

Newborn cells that are destined to become neurons, within a few hours after their genesis start to express the microtubule-associated protein doublecortin (DCX) [42]. The rate of adult hippocampal neurogenesis was determined from DCX^+^ cells present in the subgranular zone (SGZ) of the DG at two months of age as detected by immunofluorescent stainings. In *cx3cr1*^−/−^ mice, the number of DCX^+^ cells was reduced when compared to *cx3cr1*^+/+^ littermates (Fig. 1a, c). The reduction was accompanied by a decrease in the number of BrdU^+^ cells, a marker for proliferating cells, and a reduced number of BrdU^+^/DCX^+^ cells in *cx3cr1*^−/−^ mice (Fig. 1a, b). To determine, whether the lack of CX_3_CR1 had an effect on the differentiation of BrdU^+^ cells, we performed double labeling with antibodies against BrdU and the neuronal marker NeuN (Fig. 1a). Again, the number of BrdU^+^/NeuN^+^ double positive cells was reduced in *cx3cr1*^−/−^ mice compared to *cx3cr1*^+/+^ littermates (Fig. 1b), resulting in an overall lower number of NeuN^+^ cells in the DG (Fig. 1d). The reduced number of NeuN^+^ cells in the DG of *cx3cr1*^−/−^ mice had no impact on the total hippocampal volume as determined by MRI (Fig. 1e). Interestingly, DCX^+^ neuroblast proliferation and differentiation were identical in *cx3cl1*^+/+^ and *cx3cl1*^−/−^ mice (Fig. 1f-h), indicating that disturbed signaling between neuronal CX_3_CL1 and microglial CX_3_CR1 was not responsible for reduced adult neurogenesis in *cx3cr1*^−/−^ mice. In line with these findings, we observed in the adult mice, that ablation of CX_3_CR1 had no effect on microglial morphology in the SGZ, granule cell layer or hilus of the DG as displayed with three-dimensional morphometric measurements (Fig. 1j-n). We could further exclude that reduced numbers of DCX^+^ cells in *cx3cr1*^−/−^ mice were due to elevated microglial phagocytosis because there was no difference in the percentage of double positive LAMP2^+^/Iba1^+^ microglia or in the number of LAMP2^+^/Iba1^+^/DCX^+^ cells per DG in *cx3cr1*^+/+^ and *cx3cr1*^−/−^ mice (Fig. 2a, b). In addition, DCX^+^ cells showed no difference in caspase-3-mediated apoptosis as indicated by similar numbers of active-caspase-3^+^ cells per dentate gyrus in both genotypes (Fig. 2c, d).

### *Cx3cr1*^−/−^ microglia show increased sirtuin 1 and NF-kB signaling locally in the dentate gyrus

**Fig. 3:**
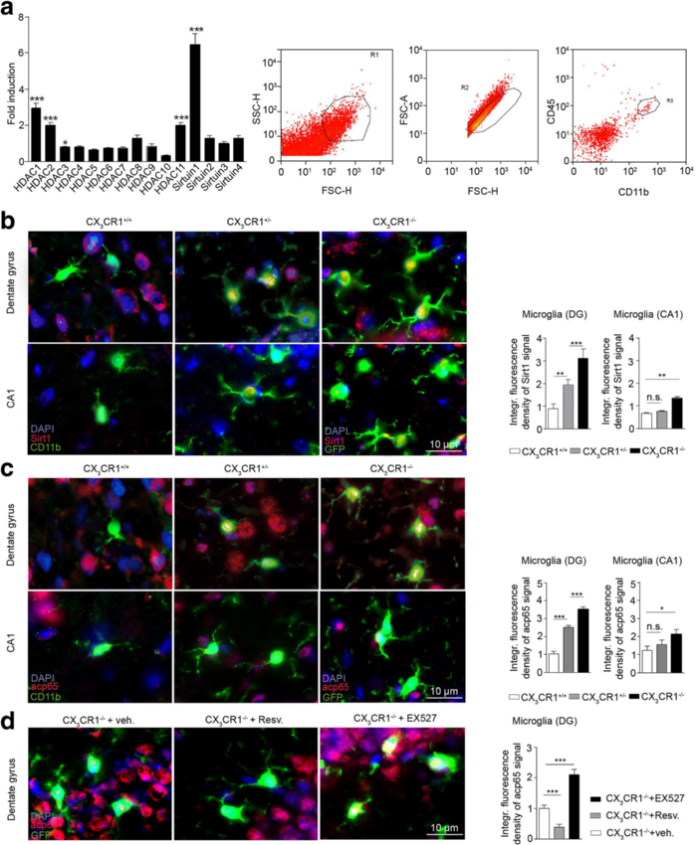
Enhanced microglial SIRT1 expression and acetylation of p65 in the DG of *cx3cr1* ^−/−^ mice. Flow cytometric analysis based on cell size and granularity identified hippocampal microglia from *cx3cr1* ^+/+^ and *cx3cr1* ^−/−^ mice with CD11b^high^/CD45^pos^ expression (**a**, FACS blots to the right). No significant induction of mRNA levels was found for HDAC4-HDAC10 and sirtuin2-sirtuin4 when *cx3cr1* ^−/−^ mRNA levels were divided by mRNA levels found in *cx3cr1* ^+/+^ microglia (*p* > 0.05, two-tailed Student’s *t*-test, *cx3cr1* ^+/+^ (*n* = 6) vs. *cx3cr1* ^−/−^ (*n* = 6)) (**a**, bar graph). HDAC1-HDAC3, HDAC11 and sirtuin1 mRNA levels were significantly higher in hippocampal microglia from *cx3cr1* ^−/−^ mice compared to *cx3cr1* ^+/+^ mice (**a**, bar graph). Bars represent normalized mRNA expression of *cx3cr1* ^−/−^ microglia (*n* = 6) in fold over *cx3cr1* ^+/+^ microglia (*n* = 6, ***p < 0.001; *p < 0.05) (**b**) Immunostaining of coronal DG (*upper row*) and CA1 (*lower row*) sections from *cx3cr1* ^+/+^ (*left*), *cx3cr1* ^+/−^ (*middle*) and *cx3cr1* ^−/−^ (*right*) mice for DAPI (*blue*), Sirt1 (*red*) and CD11b (*green*). In *cx3cr1* ^+/−^ and *cx3cr1* ^−/−^ mice microglia were labeled with GFP (*green*). In the DG area the integrated Sirt1 fluorescence signal was increased in *cx3cr1* ^+/−^ microglia compared with the signal found in *cx3cr1* ^+/+^ microglia (*p* = 0.0031, two-tailed Student’s *t*-test, *cx3cr1* ^+/+^ (*n* = 4) vs. *cx3cr1* ^+/−^ (*n* = 7)) and even more intense in *cx3cr1* ^−/−^ when compared to *cx3cr1* ^+/−^ microglia (*p* < 0.001, two-tailed Student’s *t*-test, *cx3cr1* ^+/−^ (*n* = 6) vs. *cx3cr1* ^−/−^ (*n* = 7)). In the CA1 region *cx3cr1* ^−/−^ microglial Sirt1 staining was found to be only slightly increased (*p* = 0.0021, two-tailed Student’s *t*-test, *cx3cr1* ^+/+^ (*n* = 6) vs. *cx3cr1* ^−/−^ (*n* = 6)) while there was no difference in the Sirt1 signal when comparing *cx3cr1* ^+/−^ and *cx3cr1* ^+/+^ microglia (*p* = 0.7641, two-tailed Student’s *t*-test, *cx3cr1* ^+/+^ (*n* = 6) vs. *cx3cr1* ^+/−^ (*n* = 5)). Bars represent the integrated fluorescence density of the Sirt1 signal in microglia of *cx3cr1* ^+/−^ and *cx3cr1* ^−/−^ mice relative to the Sirt1 signal in DG *cx3cr1* ^+/+^ microglia (**b**, bar graphs to the right). **c** Immunostaining for microglial acetylated p65 (*red*) in the hippocampal DG (*upper row*) and CA1 region (*lower row*) of the same mouse strains revealed the strongest signals in the DG of *cx3cr1* ^+/−^ (*p* = 0.0002, two-tailed Student’s *t*-test, *cx3cr1* ^+/+^ (*n* = 6) vs. *cx3cr1* ^+/−^ (*n* = 5)) and *cx3cr1* ^−/−^ mice (*p* = 0.0001, two-tailed Student’s *t*-test, *cx3cr1* ^+/−^ (*n* = 5) vs. *cx3cr1* ^−/−^ (*n* = 7)). There was only a mildly increased acp65 signal in CA1 microglia from *cx3cr1* ^−/−^ mice (*p* = 0.0149, two-tailed Student’s *t*-test, *cx3cr1* ^+/+^ (*n* = 5) vs. *cx3cr1* ^−/−^ (*n* = 6)). Signal intensity for microglial acp65 was indistinguishable in *cx3cr1* ^+/−^ and *cx3cr1* ^+/+^ mice (*p* = 0.1555, two-tailed Student’s *t*-test, *cx3cr1* ^+/+^ (*n* = 5) vs. *cx3cr1* ^−/−^ (*n* = 5)) (**c**, bar graphs to the right). **d** Immunostaining for acp65 in *cx3cr1* ^−/−^ microglia was significantly reduced in *cx3cr1* ^−/−^ mice pretreated with resveratrol (*p* < 0.001, two-tailed Student’s *t*-test, *cx3cr1* ^−/−^ + vehicle (*n* = 7) vs. *cx3cr1* ^−/−^ + resveratrol (*n* = 7)) and increased following EX527 treatment (*p* = 0.0002, two-tailed Student’s *t*-test, *cx3cr1* ^−/−^ + vehicle (*n* = 7) vs. *cx3cr1* ^−/−^ + EX527 (*n* = 7)). One representative experiment of two is shown. Bars represent mean ± SEM; n.s., not significant. ***p < 0.001; **p < 0.01; *p < 0.05

**Fig. 4:**
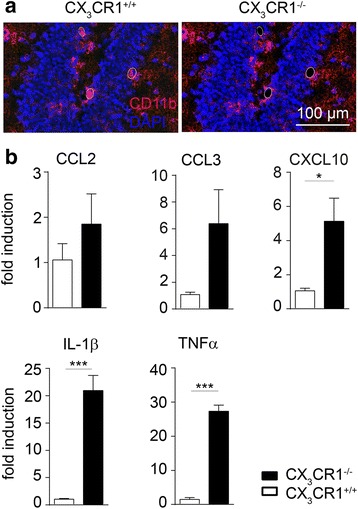
Increased cytokine expression in *cx3cr1* ^*−/−*^ microglia isolated from DG. **a** CD11b positive cells were lasermicrodissected from the DG area and RNA was isolated for real-time PCR. **b** RT-PCR experiments showed that CCL2 (*p* = 0.354, two-tailed Student’s *t*-test, *cx3cr1* ^−/−^ (*n* = 5) vs. *cx3cr1* ^+/+^ (*n* = 4)) and CCL3 (*p* = 0.107, two-tailed Student’s *t*-test, *cx3cr1* ^−/−^ (*n* = 5) vs. *cx3cr1* ^+/+^ (*n* = 4)) expression was similar in microglial RNA from *cx3cr1* ^+/+^ and *cx3cr1* ^−/−^ mice. Significantly increased expression level of CXCL10 (*p* = 0.013, two-tailed Student’s *t*-test, *cx3cr1* ^−/−^ (*n* = 5) vs. *cx3cr1* ^+/+^ (*n* = 4)), TNF-α (*p* = 0.0001, two-tailed Student’s *t*-test, *cx3cr1* ^−/−^ (*n* = 5) vs. *cx3cr1* ^+/+^ (*n* = 4)) and IL-1β (*p* = 0.0004, two-tailed Student’s *t*-test, *cx3cr1* ^−/−^ (*n* = 5) vs. *cx3cr1* ^+/+^ (*n* = 4)) mRNA were detected in *cx3cr1* ^−/−^ mice. One representative experiment of two is shown. Bars represent mean ± SEM; n.s., not significant. ***p < 0.001; *p < 0.05

CX_3_CR1 is a G_i_-protein coupled receptor, which inhibits cAMP signaling including the cAMP-dependent protein kinase A (PKA) [43]. All mammalian histone deacetylases (HDACs) possess potential phosphorylation sites and some of them can be phosphorylated by PKA [44]. The phosphorylation status of HDACs often determines the degree of transcriptional regulation of their target genes including their own expression in a negative feedback loop as described for HDAC1 [45]. Thus, we set out to perform a quantitative RT-PCR analysis for expression of HDACs in microglia obtained by fluorescence activated cell sorting (FACS) from hippocampi of *cx3cr1*^−/−^ and *cx3cr1*^+/+^ mice (Fig. 3a). RT-PCR results revealed that, from all HDACs analyzed, sirtuin 1 (SIRT1) was highest expressed in *cx3cr1*^−/−^ microglia when compared to microglia isolated from *cx3cr1*^+/+^ mice (Fig. 3a). When hippocampal brain sections were stained with antibodies against SIRT1, the strongest signal was detected in microglia (GFP^+^) from *cx3cr1*^−/−^ mice in the DG, with a gradual decrease in SIRT1 signal strength, when microglia from *cx3cr1*^+/−^ (GFP^+^) and *cx3cr1*^+/+^ mice (CD11b^+^) were analyzed (Fig. 3b, upper row). Microglial SIRT1 signals were diminished in the hippocampal CA1 area for all three genotypes. Still, *cx3cr1*^−/−^ microglia showed the most intense SIRT1 signal when compared to microglia from heterozygous and wildtype littermates (Fig. 3b, lower row). Interestingly, the elevated integrated fluorescence density of the SIRT1 signal in *cx3cr1*^−/−^ microglia was paralleled by a high signal intensity of acetylated p65 (acp65) in *cx3cr1*^−/−^ microglia within the DG (Fig. 3c, upper row) and the CA1 region (Fig. 3c, lower row), indicative of an active NF-kB complex [46]. In both areas, the acp65 signal was diminished in microglia from *cx3cr1*^+/−^ and *cx3cr1*^+/+^ mice and was overall less intense in microglia located in the hippocampal CA1 region when compared to DG microglia (Fig. 3c). When *cx3cr1*^−/−^ mice were treated with resveratrol, a SIRT1 activator [47], the acp65 signal in microglia within the DG was reduced. On the other hand, the acp65 signal in *cx3cr1*^−/−^ mice was amplified by treating the animals with EX527, a selective inhibitor of SIRT1 [48] (Fig. 3d). Since cytokines are largely induced through the NF-kB pathway we isolated microglia via laser-microdissection from the DG area (Fig. 4a) and found elevated level of CXCL10, TNFα and IL-β mRNA in *cx3cr1*^*−/−*^ microglia while expression of CCL2 and CCL3 showed no difference when compared to *cx3cr1*^*+/+*^ microglia mRNA (Fig. 4b).

### Activation of sirtuin 1 restores cognitive performance and neurogenesis in *cx3cr1*^−/−^ mice

**Fig. 5:**
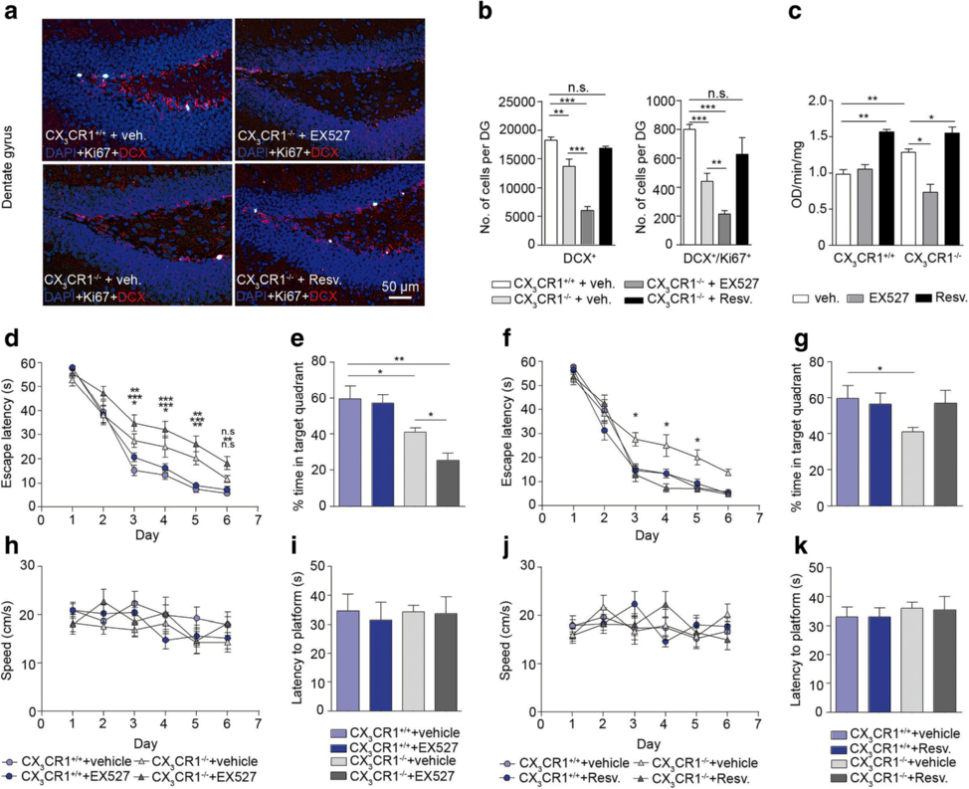
Activation of microglial SIRT1 increases proliferation of DCX^+^ cells in *cx3cr1* ^−/−^ mice and improves spatial learning and memory. **a** Immunohistochemistry in DG reveals cell nuclei stained with DAPI (*blue*), cells positive for the proliferation marker Ki67 (*white*) and positive for DCX (*red*) in vehicle-treated *cx3cr1* ^+/+^ (*top, left*) and *cx3cr1* ^−/−^ (*bottom, left*) mice and in *cx3cr1* ^−/−^ mice treated with EX527 (*top, right*) or resveratrol (*bottom, right*). **b** Numbers of DCX^+^ (*p* = 0.0078, two-tailed Student’s *t*-test, *cx3cr1* ^+/+^ + vehicle (*n* = 6) vs. *cx3cr1* ^−/−^ + vehicle (*n* = 6)) and DCX^+^/Ki67^+^ (*p* = 0.0003, two-tailed Student’s *t*-test, *cx3cr1* ^+/+^ + vehicle (*n* = 6) vs. *cx3cr1* ^−/−^ + vehicle (*n* = 6)) cells were reduced in *cx3cr1* ^−/−^ mice compared with *cx3cr1* ^+/+^ mice. Both groups were vehicle-treated. A further reduction of DCX^+^ (*p* = 0.0003, two-tailed Student’s *t*-test, *cx3cr1* ^−/−^ + vehicle (*n* = 6) vs. *cx3cr1* ^−/−^ + EX527 (*n* = 6)) and DCX^+^/Ki67^+^ (*p* = 0.0041, two-tailed Student’s *t*-test, *cx3cr1* ^−/−^ + vehicle (*n* = 6) vs. *cx3cr1* ^−/−^ + EX527 (*n* = 6)) cells occurred when *cx3cr1* ^−/−^ mice were pretreated with EX527. There was no difference in cell numbers of DCX^+^ (*p* = 0.0597, two-tailed Student’s *t*-test, *cx3cr1* ^−/−^ + Resv. (*n* = 6)) and DCX^+^/Ki67^+^ (*p* = 0.184, two-tailed Student’s *t*-test, *cx3cr1* ^−/−^ + Resv. (*n* = 6)) cells in vehicle-treated *cx3cr1* ^+/+^ and resveratrol-treated *cx3cr1* ^−/−^ mice. **c** A sirtuin 1 activity assay using nuclear extracts from sorted microglia revealed significant differences between vehicle-treated *cx3cr1* ^+/+^ (*n* = 6) and *cx3cr1* ^−/−^ mice (*n* = 6) (*p* = 0.0029, two-tailed Student’s *t*-test), vehicle-treated *cx3cr1* ^+/+^ and resveratrol-treated *cx3cr1* ^+/+^ mice (*n* = 6) (*p* = 0.0013, two-tailed Student’s *t*-test), vehicle-treated *cx3cr1* ^−/−^ and EX527-treated *cx3cr1* ^−/−^ mice (*n* = 7) (*p* = 0.02, two-tailed Student’s *t*-test) and between vehicle-treated *cx3cr1* ^−/−^ and resveratrol-treated *cx3cr1* ^−/−^ mice (*n* = 6) (*p* = 0.02, two-tailed Student’s *t*-test).**d**, **f** Learning abilities as measured by escape latency to find the hidden platform over 6 days (two-way ANOVA followed by Bonferroni *post hoc*, *p* < 0.05, F_1,50_ = 44.28, *p* < 0.0001, *cx3cr1* ^+/+^ + vehicle (*n* = 6) vs. *cx3cr1* ^−/−^ + EX527 (*n* = 7), *F*_1,46_ = 12,34, *p* = 0.001, *cx3cr1* ^+/+^ + vehicle (*n* = 6) vs. *cx3cr1* ^−/−^ + vehicle (*n* = 6), *F*_1,46_ = 2.07, *p* = 0.1565, *cx3cr1* ^+/+^ + vehicle (*n* = 6) vs. *cx3cr1* ^+/+^ + EX527 (*n* = 6). **e**, **g** Spatial memory retention evaluated during the probe test performed 24 h later (two-tailed Student’s *t*-test, *p* = 0.0343, *cx3cr1* ^+/+^ + vehicle (*n* = 6) vs. *cx3cr1* ^−/−^ + vehicle (*n* = 6); *p* = 0.0119, *cx3cr1* ^−/−^ + vehicle (*n* = 6) vs. *cx3cr1* ^−/−^ + EX527 (*n* = 6); *p* = 0.0035, *cx3cr1* ^+/+^ + vehicle (*n* = 6) vs. *cx3cr1* ^−/−^ + EX527 (*n* = 6)), **h**,**j** swim speed during the learning phase and **i**, **k** latency to reach the visible platform of *cx3cr1* ^+/+^ and *cx3cr1* ^−/−^ pre-treated with either vehicle alone or with vehicle in combination with EX527 or resveratrol. One representative experiment of three is shown. Bars represent mean ± SEM; n.s., not significant. Asterisks depict *p* value when compared to *cx3cr1* ^+/+^+ vehicle group. ***p < 0.001; **p < 0.01; *p < 0.05

With the help of a sirtuin 1 activity assay we could determine that the elevated level of SIRT1 in hippocampal *cx3cr1*^−/−^ microglia resulted in increased SIRT1 activity in comparison to SIRT1 activity in microglia from *cx3cr1*^+/+^ hippocampi (Fig. 5c). In order to determine whether modulation of SIRT1 activity affects the number and proliferation of DCX^+^ cells in the DG, as indicated by double-staining with the cell proliferation marker Ki67, we treated *cx3cr1*^−/−^ mice with either EX527 or with resveratrol. While the treatment with EX527 further reduced neuronal lineage proliferation and the number of DCX^+^ cells when compared to vehicle-treatment, activation of SIRT1 by resveratrol recovered proliferation and the number of DCX^+^ cells in *cx3cr1*^−/−^ mice returned back to the situation found in *cx3cr1*^+/+^ mice (Fig. 5a, b). The SIRT1 activity assay showed that EX527 efficiently reduced SIRT1 activity in hippocampal microglia from *cx3cr1*^−/−^ mice and that resveratrol activated SIRT1 even above the elevated SIRT1 activity found in vehicle-treated *cx3cr1*^−/−^ mice (Fig. 5c). To study the impact of SIRT1 activity and changes in adult neurogenesis on spatial learning and memory, animals were subjected to the Morris Water Maze test. The treatment of *cx3cr1*^+/+^ mice with EX527 had no effect on either escape latency during the learning phase or time spent in the target quadrant as an indicator of memory (Fig. 5d, e). This finding suggests that SIRT1 activity is not involved in spatial learning and memory under baseline conditions. In contrast, *cx3cr1*^−/−^ mice showed impaired learning and memory when compared to *cx3cr1*^+/+^ mice (Fig. 5d, e). This impairment became even more prominent in *cx3cr1*^−/−^ mice pretreated with EX527 (Fig. 5d, e). *cx3cr1*^+/+^ and *cx3cr1*^−/−^ mice showed similar swim speed (Fig. 5h) and latency to find the visible platform (Fig. 5i) after pretreatment with vehicle alone or in combination with EX527. Activation of SIRT1 by injection of resveratrol improved spatial learning and memory in *cx3cr1*^−/−^ mice up to the level demonstrated by *cx3cr1*^+/+^ mice (Fig. 5f, g). Again, swim speed and time to reach the visible platform were not affected by the treatment indicating that mice were not visually or motivationally impaired (Fig. 5j, k).

## Discussion

With the present study we show that, under resting conditions, SIRT1 and the NF-kB pathway are activated in *cx3cr1*^−/−^ microglia residing within the murine DG area. This activation seems to be restricted to the DG and was largely diminished in the hippocampal CA1 region. As a result of pharmacological SIRT1 activation, impaired adult neurogenesis and lowered hippocampal cognitive performance was restored in *cx3cr1*^−/−^ mice.

We hypothesize that the NF-kB signaling pathway and SIRT1 enzyme in microglia interact to maintain cellular homeostasis in vivo. Since the fractalkine receptor CX_3_CR1 inhibits cAMP signaling including the cAMP-dependent protein kinase A (PKA) via coupling to a G_i_-protein coupled receptor [43], deletion of CX_3_CR1 from microglia might facilitate activation of PKA and subsequently NF-kB activation [49]. Stimuli causing PKA activation appear to be restricted to certain brain regions with e.g. enhanced cellular turnover like the DG because only marginal acp65 signals were detected outside the DG as seen in the CA1 area. In response to increased acetylation of p65 in *cx3cr1*^−/−^ microglia, SIRT1 activity is amplified, most likely to counteract excessive NF-kB signaling. Activation of SIRT1 can induce deacetylation of the RelA/p65 component of the NF-kB complex. The deacetylation of Lys310 inhibits the transactivation capacity of RelA/p65 subunit and consequently suppresses the transcription of the NF-kB-dependent gene expression [20]. However, in *cx3cr1*^−/−^ microglia SIRT1 activity, although elevated, appears to be insufficient to prevent NF-kB-dependent gene expression [50] as indicated by elevated protein levels of IL-1β in the hippocampus [13] or by increased CXCL10, TNF-α and IL-1β mRNA expression in microglia micro-dissected from DG of *cx3cr1*^−/−^ mice (Fig. 4). Only additional SIRT1 activation can effectively counteract activation of the NF-kB pathway. Interestingly, in wild-type mice where no microglial NF-kB activation was detectable, activation of SIRT1 had no effect on adult neurogenesis or performance in the water maze test (data not shown). Previous studies have shown that especially IL-1β can impair adult neurogenesis by decreasing proliferation in the DG of *cx3cr1*^−/−^ mice [13]. A similar mechanism was observed in wild-type mice under stressed conditions when elevated IL-1β reduced hippocampal neurogenesis and administration of an IL-1 receptor antagonist restored the neurogenesis rate following stress exposure [51]. Notably, despite the pro-inflammatory activation status of *cx3cr1*^−/−^ microglia, no morphological changes could be detected in comparison to *cx3cr1*^+/+^ microglia, confirming previous findings within an independent set of experiments [52]. During development in the hippocampus, microglial morphology seems to depend, at least partially, on the fractalkine receptor considering that at P8 a small population of *cx3cr1*^−/+^ cells was typified by a very large surface area. This group of cells was absent in brains of *cx3cr1*^−/−^ mice [53]. However, one report based on immunohistochemistry indicates that under normal physiological conditions genetic *cx3cr1* deficiency is associated with microglial alterations, including increased cell number and enlargement of the soma [54]. While the receptor for CX_3_CL1, CX_3_CR1, is highly expressed on microglia, CX_3_CL1 is constitutively expressed at high levels on healthy neurons. CX_3_CL1 is expressed as a transmembrane protein that can be cleaved in a soluble form, consisting of the extracellular N-terminal chemokine domain, by the activity of the lysosomal cysteine protease cathepsin S (CatS) or by members of the disintegrin and metalloproteinase (ADAM) family, ADAM-10 and ADAM-17 [55]. The anatomical expression of CX_3_CL1 on neurons and CX_3_CR1 on microglia suggests that neurons may maintain microglia in a surveilling/ramified state through a repressive CX_3_CL1 signal [56]. From this point of view it is unexpected to find intact proliferation of neural stem/progenitor cells and adult neurogenesis in CX_3_CL1-deficient mice while the same process is impaired in mice lacking CX_3_CR1. If binding of CX_3_CL1 elicits a tonic inhibitory signal, which maintains microglia in a quiescent state, its absence should result in activated or pro-inflammatory microglia with negative impact on neurogenesis and neurodevelopment. While there is a consensus among studies for a neuroprotective role of CX_3_CL1 signaling in vitro*,* some in vivo studies even suggest a neurotoxic role of CX_3_CL1 as seen in animal models for Alzheimer’s- and Parkinson’s disease. Here, CX_3_CL1 can act as a repressor of microglial phagocytic activity and cause overall microglia activation [57, 58]. A further remarkable mode of action was observed in lung endothelial cells, which respond to stimuli and produce CX_3_CL1. This leads to the endothelial attachment of the subset mononuclear leukocytes that express the sole CX_3_CL1 receptor, CX_3_CR1 [59]. Following a challenge with lipopolysaccharides (LPS), *cx3cl1*^−/−^ mice exhibit reduced expression of CX_3_CR1 and impaired NF-kB signaling in lung tissue when compared to wt controls [60]. CX_3_CL1 is a molecule that may have various activities, with either no, beneficial or destructive potential, likely depending on the activation state of its main target cells. In support of our findings *cx3cl1*^−/−^ mice do not have histologic abnormalities in any major organs (including the brain), hematopoietic lineages in blood and lymphoid tissue are essentially normal, and they do not exhibit any overt behavioral abnormalities [21]. There is also the possibility of an alternative CX_3_CR1 ligand, which might act similar to CX_3_CL1. In humans eotaxin-3/CC chemokine ligand 26 was recently reported to be a functional ligand for CX_3_CR1 [61]; in mice, the *CCL26* gene, however, may be a pseudogene since no cDNA or expressed sequence tag (EST) has been reported [62]. Similar to previous reports we found that hippocampal neurogenesis was decreased in mice that lack CX_3_CR1 [15]. These mice display significant deficits in cognitive functions and LTP induction due to increased action of IL-1β [14]. There are two reports indicating that *cx3cr1*^−/−^ mice have improved hippocampal cognitive abilities compared to wild-type controls [15, 54] with either enhanced [54] or impaired [15] generation of neuronal precursors. The reason for this inconsistency is presently unclear.

## Conclusions

Our findings indicate that in the SGZ of the DG area in *cx3cr1*^−/−^ mice the number of DCX^+^ cells is reduced, independent of the CX_3_CR1 ligand CX_3_CL1. Enhanced microglial NF-kB-dependent gene expression in the DG results in elevated levels of chemokines such as IL-1β and consequently in the inhibition of neurogenesis and spatial cognitive function. Manipulation of SIRT1 activity interferes with NF-kB signaling, adult neurogenesis and ultimately hippocampal learning and memory.

## Abbreviations

EGFP: Enhanced green fluorescent protein
FACS: Fluorescence activated cell sorting
HDAC: Histone deacetylase
LTP: Long-term potentiation
MWM: Morris water maze
NF-kB: Nuclear factor kappa-light-chain-enhancer of activated B cells
SIRT1: Sirtuin 1

## References

[1] Al-Aoukaty A, Rolstad B, Giaid A, Maghazachi AA (1998). MIP-3alpha, MIP-3beta and fractalkine induce the locomotion and the mobilization of intracellular calcium, and activate the heterotrimeric G proteins in human natural killer cells. Immunology.

[2] Jung S, Aliberti J, Graemmel P, Sunshine MJ, Kreutzberg GW, Sher A, Littman DR (2000). Analysis of fractalkine receptor CX(3)CR1 function by targeted deletion and green fluorescent protein reporter gene insertion. Mol Cell Biol.

[3] Ginhoux F, Greter M, Leboeuf M, Nandi S, See P, Gokhan S, Mehler MF, Conway SJ, Ng LG, Stanley ER (2010). Fate mapping analysis reveals that adult microglia derive from primitive macrophages. Science.

[4] Kierdorf K, Erny D, Goldmann T, Sander V, Schulz C, Perdiguero EG, Wieghofer P, Heinrich A, Riemke P, Holscher C (2013). Microglia emerge from erythromyeloid precursors via Pu.1- and Irf8-dependent pathways. Nat Neurosci.

[5] Schulz C, Gomez Perdiguero E, Chorro L, Szabo-Rogers H, Cagnard N, Kierdorf K, Prinz M, Wu B, Jacobsen SE, Pollard JW (2012). A lineage of myeloid cells independent of Myb and hematopoietic stem cells. Science.

[6] Shemer A, Erny D, Jung S, Prinz M (2015). Microglia plasticity during health and disease: an immunological perspective. Trends Immunol.

[7] Paolicelli RC, Bolasco G, Pagani F, Maggi L, Scianni M, Panzanelli P, Giustetto M, Ferreira TA, Guiducci E, Dumas L (2011). Synaptic pruning by microglia is necessary for normal brain development. Science.

[8] Schafer DP, Lehrman EK, Kautzman AG, Koyama R, Mardinly AR, Yamasaki R, Ransohoff RM, Greenberg ME, Barres BA, Stevens B (2012). Microglia sculpt postnatal neural circuits in an activity and complement-dependent manner. Neuron.

[9] Hatori K, Nagai A, Heisel R, Ryu JK, Kim SU (2002). Fractalkine and fractalkine receptors in human neurons and glial cells. J Neurosci Res.

[10] Kim KW, Vallon-Eberhard A, Zigmond E, Farache J, Shezen E, Shakhar G, Ludwig A, Lira SA, Jung S (2011). In vivo structure/function and expression analysis of the CX3C chemokine fractalkine. Blood.

[11] Chapman GA, Moores K, Harrison D, Campbell CA, Stewart BR, Strijbos PJ (2000). Fractalkine cleavage from neuronal membranes represents an acute event in the inflammatory response to excitotoxic brain damage. J Neurosci.

[12] Haskell CA, Cleary MD, Charo IF (1999). Molecular uncoupling of fractalkine-mediated cell adhesion and signal transduction. Rapid flow arrest of CX3CR1-expressing cells is independent of G-protein activation. J Biol Chem.

[13] Bachstetter AD, Morganti JM, Jernberg J, Schlunk A, Mitchell SH, Brewster KW, Hudson CE, Cole MJ, Harrison JK, Bickford PC (2011). Fractalkine and CX 3 CR1 regulate hippocampal neurogenesis in adult and aged rats. Neurobiol Aging.

[14] Rogers JT, Morganti JM, Bachstetter AD, Hudson CE, Peters MM, Grimmig BA, Weeber EJ, Bickford PC, Gemma C (2011). CX3CR1 deficiency leads to impairment of hippocampal cognitive function and synaptic plasticity. J Neurosci.

[15] Maggi L, Scianni M, Branchi I, D’Andrea I, Lauro C, Limatola C (2011). CX(3)CR1 deficiency alters hippocampal-dependent plasticity phenomena blunting the effects of enriched environment. Front Cell Neurosci.

[16] Xue J, Dong JH, Huang GD, Qu XF, Wu G, Dong XR (2014). NF-kappaB signaling modulates radiationinduced microglial activation. Oncol Rep.

[17] Hickman SE, Kingery ND, Ohsumi TK, Borowsky ML, Wang LC, Means TK, El Khoury J (2013). The microglial sensome revealed by direct RNA sequencing. Nat Neurosci.

[18] Benzing WC, Wujek JR, Ward EK, Shaffer D, Ashe KH, Younkin SG, Brunden KR (1999). Evidence for glial-mediated inflammation in aged APP(SW) transgenic mice. Neurobiol Aging.

[19] Chen J, Zhou Y, Mueller-Steiner S, Chen LF, Kwon H, Yi S, Mucke L, Gan L (2005). SIRT1 protects against microglia-dependent amyloid-beta toxicity through inhibiting NF-kappaB signaling. J Biol Chem.

[20] Yeung F, Hoberg JE, Ramsey CS, Keller MD, Jones DR, Frye RA, Mayo MW (2004). Modulation of NF-kappaB-dependent transcription and cell survival by the SIRT1 deacetylase. EMBO J.

[21] Cook DN, Chen SC, Sullivan LM, Manfra DJ, Wiekowski MT, Prosser DM, Vassileva G, Lira SA (2001). Generation and analysis of mice lacking the chemokine fractalkine. Mol Cell Biol.

[22] Morris R (1984). Developments of a water-maze procedure for studying spatial learning in the rat. J Neurosci Methods.

[23] Kumar R, Hunt CR, Gupta A, Nannepaga S, Pandita RK, Shay JW, Bachoo R, Ludwig T, Burns DK, Pandita TK (2011). Purkinje cell-specific males absent on the first (mMof) gene deletion results in an ataxia-telangiectasia-like neurological phenotype and backward walking in mice. Proc Natl Acad Sci U S A.

[24] Park HR, Kong KH, Yu BP, Mattson MP, Lee J (2012). Resveratrol inhibits the proliferation of neural progenitor cells and hippocampal neurogenesis. J Biol Chem.

[25] Darcy MJ, Trouche S, Jin SX, Feig LA (2014). Age-dependent role for Ras-GRF1 in the late stages of adult neurogenesis in the dentate gyrus. Hippocampus.

[26] Dann A, Poeck H, Croxford AL, Gaupp S, Kierdorf K, Knust M, Pfeifer D, Maihoefer C, Endres S, Kalinke U (2012). Cytosolic RIG-I-like helicases act as negative regulators of sterile inflammation in the CNS. Nat Neurosci.

[27] Mildner A, Schmidt H, Nitsche M, Merkler D, Hanisch UK, Mack M, Heikenwalder M, Bruck W, Priller J, Prinz M (2007). Microglia in the adult brain arise from Ly-6ChiCCR2+ monocytes only under defined host conditions. Nat Neurosci.

[28] Goldmann T, Wieghofer P, Muller PF, Wolf Y, Varol D, Yona S, Brendecke SM, Kierdorf K, Staszewski O, Datta M (2013). A new type of microglia gene targeting shows TAK1 to be pivotal in CNS autoimmune inflammation. Nat Neurosci.

[29] Raasch J, Zeller N, van Loo G, Merkler D, Mildner A, Erny D, Knobeloch KP, Bethea JR, Waisman A, Knust M (2011). IkappaB kinase 2 determines oligodendrocyte loss by non-cell-autonomous activation of NF-kappaB in the central nervous system. Brain.

[30] Fatt MP, Cancino GI, Miller FD, Kaplan DR (2014). p63 and p73 coordinate p53 function to determine the balance between survival, cell death, and senescence in adult neural precursor cells. Cell Death Differ.

[31] Chal J, Oginuma M, Al Tanoury Z, Gobert B, Sumara O, Hick A, Bousson F, Zidouni Y, Mursch C, Moncuquet P (2015). Differentiation of pluripotent stem cells to muscle fiber to model Duchenne muscular dystrophy. Nat Biotechnol.

[32] Fulmer CG, VonDran MW, Stillman AA, Huang Y, Hempstead BL, Dreyfus CF (2014). Astrocyte-derived BDNF supports myelin protein synthesis after cuprizone-induced demyelination. J Neurosci.

[33] Farioli-Vecchioli S, Micheli L, Saraulli D, Ceccarelli M, Cannas S, Scardigli R, Leonardi L, Cina I, Costanzi M, Ciotti MT (2012). Btg1 is required to maintain the pool of stem and progenitor cells of the dentate gyrus and subventricular zone. Front Neurosci.

[34] Nistico R, Florenzano F, Mango D, Ferraina C, Grilli M, Di Prisco S, Nobili A, Saccucci S, D’Amelio M, Morbin M (2015). Presynaptic c-Jun N-terminal Kinase 2 regulates NMDA receptor-dependent glutamate release. Sci Rep.

[35] Gould E, Beylin A, Tanapat P, Reeves A, Shors TJ (1999). Learning enhances adult neurogenesis in the hippocampal formation. Nat Neurosci.

[36] Mildner A, Schlevogt B, Kierdorf K, Bottcher C, Erny D, Kummer MP, Quinn M, Bruck W, Bechmann I, Heneka MT (2011). Distinct and non-redundant roles of microglia and myeloid subsets in mouse models of Alzheimer’s disease. J Neurosci.

[37] West MJ, Slomianka L, Gundersen HJ (1991). Unbiased stereological estimation of the total number of neurons in thesubdivisions of the rat hippocampus using the optical fractionator. Anat Rec.

[38] Goldmann T, Zeller N, Raasch J, Kierdorf K, Frenzel K, Ketscher L, Basters A, Staszewski O, Brendecke SM, Spiess A (2015). USP18 lack in microglia causes destructive interferonopathy of the mouse brain. EMBO J.

[39] Harsan LA, David C, Reisert M, Schnell S, Hennig J, von Elverfeldt D, Staiger JF (2013). Mapping remodeling of thalamocortical projections in the living reeler mouse brain by diffusion tractography. Proc Natl Acad Sci U S A.

[40] Harsan LA, Paul D, Schnell S, Kreher BW, Hennig J, Staiger JF, von Elverfeldt D (2010). In vivo diffusion tensor magnetic resonance imaging and fiber tracking of the mouse brain. NMR Biomed.

[41] Kumar P, Periyasamy R, Das S, Neerukonda S, Mani I, Pandey KN (2014). All-trans retinoic acid and sodium butyrate enhance natriuretic peptide receptor a gene transcription: role of histone modification. Mol Pharmacol.

[42] Brown JP, Couillard-Despres S, Cooper-Kuhn CM, Winkler J, Aigner L, Kuhn HG (2003). Transient expression of doublecortin during adult neurogenesis. J Comp Neurol.

[43] Combadiere C, Salzwedel K, Smith ED, Tiffany HL, Berger EA, Murphy PM (1998). Identification of CX3CR1. A chemotactic receptor for the human CX3C chemokine fractalkine and a fusion coreceptor for HIV-1. J Biol Chem.

[44] Sengupta N, Seto E (2004). Regulation of histone deacetylase activities. J Cell Biochem.

[45] Zupkovitz G, Tischler J, Posch M, Sadzak I, Ramsauer K, Egger G, Grausenburger R, Schweifer N, Chiocca S, Decker T (2006). Negative and positive regulation of gene expression by mouse histone deacetylase 1. Mol Cell Biol.

[46] Chen LF, Mu Y, Greene WC (2002). Acetylation of RelA at discrete sites regulates distinct nuclear functions of NF-kappaB. EMBO J.

[47] Cao D, Wang M, Qiu X, Liu D, Jiang H, Yang N, Xu RM (2015). Structural basis for allosteric, substrate-dependent stimulation of SIRT1 activity by resveratrol. Genes Dev.

[48] Solomon JM, Pasupuleti R, Xu L, McDonagh T, Curtis R, DiStefano PS, Huber LJ (2006). Inhibition of SIRT1 catalytic activity increases p53 acetylation but does not alter cell survival following DNA damage. Mol Cell Biol.

[49] Min KJ, Yang MS, Jou I, Joe EH (2004). Protein kinase A mediates microglial activation induced by plasminogen and gangliosides. Exp Mol Med.

[50] Liu TF, Yoza BK, El Gazzar M, Vachharajani VT, McCall CE (2011). NAD + −dependent SIRT1 deacetylase participates in epigenetic reprogramming during endotoxin tolerance. J Biol Chem.

[51] Koo JW, Duman RS (2008). IL-1beta is an essential mediator of the antineurogenic and anhedonic effects of stress. Proc Natl Acad Sci U S A.

[52] Hellwig S, Brioschi S, Dieni S, Frings L, Masuch A, Blank T, Biber K. Altered microglia morphology and higher resilience to stress-induced depression-like behavior in CX3CR1-deficient mice. Brain Behav Immun. 2015. Doi 10.1016/j.bbi.2015.11.008 10.1016/j.bbi.2015.11.008 26576722

[53] Pagani F, Paolicelli RC, Murana E, Cortese B, Di Angelantonio S, Zurolo E, Guiducci E, Ferreira TA, Garofalo S, Catalano M (2015). Defective microglial development in the hippocampus of Cx3cr1 deficient mice. Front Cell Neurosci.

[54] Reshef R, Kreisel T, Beroukhim Kay D, Yirmiya R (2014). Microglia and their CX3CR1 signaling are involved in hippocampal- but not olfactory bulb-related memory and neurogenesis. Brain Behav Immun.

[55] Hundhausen C, Misztela D, Berkhout TA, Broadway N, Saftig P, Reiss K, Hartmann D, Fahrenholz F, Postina R, Matthews V (2003). The disintegrin-like metalloproteinase ADAM10 is involved in constitutive cleavage of CX3CL1 (fractalkine) and regulates CX3CL1-mediated cell-cell adhesion. Blood.

[56] Ransohoff RM, Liu L, Cardona AE (2007). Chemokines and chemokine receptors: multipurpose players in neuroinflammation. Int Rev Neurobiol.

[57] Liu Z, Condello C, Schain A, Harb R, Grutzendler J (2010). CX3CR1 in microglia regulates brain amyloid deposition through selective protofibrillar amyloid-beta phagocytosis. J Neurosci.

[58] Shan S, Hong-Min T, Yi F, Jun-Peng G, Yue F, Yan-Hong T, Yun-Ke Y, Wen-Wei L, Xiang-Yu W, Jun M (2011). New evidences for fractalkine/CX3CL1 involved in substantia nigral microglial activation and behavioral changes in a rat model of Parkinson’s disease. Neurobiol Aging.

[59] Cambien B, Pomeranz M, Schmid-Antomarchi H, Millet MA, Breittmayer V, Rossi B, Schmid-Alliana A (2001). Signal transduction pathways involved in soluble fractalkine-induced monocytic cell adhesion. Blood.

[60] Ding XM, Pan L, Wang Y, Xu QZ. Baicalin exerts protective effects against lipopolysaccharide-induced acute lung injury by regulating the crosstalk between the CX3CL1-CX3CR1 axis and NF-kappaB pathway in CX3CL1-knockout mice. Int J Mol Med. 2016. Doi 10.3892/ijmm.2016.2456 10.3892/ijmm.2016.2456 PMC4771101 26782291

[61] Nakayama T, Watanabe Y, Oiso N, Higuchi T, Shigeta A, Mizuguchi N, Katou F, Hashimoto K, Kawada A, Yoshie O (2010). Eotaxin-3/CC chemokine ligand 26 is a functional ligand for CX3CR1. J Immunol.

[62] Pope SM, Fulkerson PC, Blanchard C, Akei HS, Nikolaidis NM, Zimmermann N, Molkentin JD, Rothenberg ME (2005). Identification of a cooperative mechanism involving interleukin-13 and eotaxin-2 in experimental allergic lung inflammation. J Biol Chem.

